# Monolayered Platinum Nanoparticles as Efficient Electrocatalysts for the Mass Production of Electrolyzed Hydrogen Water

**DOI:** 10.1038/s41598-020-67107-1

**Published:** 2020-06-23

**Authors:** Yanqing Wang, Bunshi Fugetsu, Ichiro Sakata, Chika Fujisue, Shigeru Kabayama, Norio Tahara, Shinkatsu Morisawa

**Affiliations:** 1 College of Polymer Science and Engineering, Sichuan University, Chengdu, 610065 China; 2 Nihon Trim Co. Ltd, Oyodonaka, Kita-ku, Osaka, Japan; 3 Institute for Future Initiatives, The University of Tokyo, Bunkyo-ku, Tokyo, 113-0032 Japan; 4 School of Engineering, The University of Tokyo, Bunkyo-ku, Tokyo, 113-0032 Japan

**Keywords:** Biotechnology, Electrocatalysis

## Abstract

High-performance/low-cost platinum (Pt)-based electrocatalysts have been established by top-coating both sides of a titanium plate with Pt nanoparticles. The average diameter of the Pt nanoparticles used in this study is approximately 100 nm. Three types of Pt top-coated Pt/Ti electrocatalysts, each having different top-coated Pt layer thicknesses, are prepared. Type I is a monolayered Pt top-coated type, in which the thickness of the top-coated Pt layer is approximately 100 nm; Type II is a few-layered type with a top-coated Pt layer thickness of approximately 200 nm, and Type III is a multilayered type with a top-coated Pt layer thickness of approximately 750 nm. The mass loading of Pt is 0.0215 mg cm^−2^ for Type I, 0.043 mg cm^−2^ for Type II, and 0.161 mg cm^−2^ for Type III. The electrocatalytic activities of each type of Pt/Ti electrocatalyst are evaluated through the electrolysis of acidic water and tap water. Type I gives the highest electrocatalytic efficiencies, which are comparable or even better than the electrocatalytic efficiencies of the state-of-the-art commercially available Pt/C electrode and other metal-/carbon-based HER catalysts. For example, in the case of the electrolysis of acidic water at an overpotential of 0.15 V, Type I shows a Tafel slope of 29 mV dec^−1^ and a current density of 27.5 mA cm^−2^. Even in the case of the electrolysis of tap water, Type I gives an HER Faradaic efficiency of 92%. A model of water (H_2_O), hydronium ions (H_3_O^+^), and hydroxyl ions (OH^−^) properly adsorbing on the Pt (111) facet is proposed to explain the electrocatalytic mechanism. New insights into the distinguishing properties of the resultant electrolyzed hydrogen water (EHW), namely, the healthy beneficial effects of EHW, are also described, and a new concept of storing and carrying reductive hydrogen (H*) by free Pt nanoparticles is proposed.

## Introduction

Electrolyzed hydrogen water (EHW) is capable of improving gastrointestinal functionalities. This medical application of EHW was specified officially for the first time in 1965 by the Ministry of Health, Labour and Welfare of Japan^1^; currently, EHW is recognized national wide in Japan. Additional health benefits, such as antidiabetic effects^2,3^, antiaging effects^4,5^, anticancer effects^6–10^, anti-arteriosclerosis effects^11^, anti-inflammation effects^12^ and anti-neurodegenerative effects^13^, have also been reported for EHW soon after physiological studies were performed. Platinum (Pt) has entirely been utilized as the electrocatalyst for the electrolysis of EHW. Certain amounts of Pt clusters and/or Pt nanoparticles are released from the Pt-based electrocatalysts (electrodes) during the electrolysis of EHW^4^. These free Pt clusters and/or Pt nanoparticles are capable of converting hydrogen molecules (H_2_) into reductive hydrogen species (H·) via Pt/H_2_ catalytic interactions^14,15^, thus introducing distinguishing reductive properties to EHW.

Performance vs. cost has always been the topmost priority encountered in Pt/H_2_ industries^16^. In 2005, General Motors (GM) Corporation reported a record Pt cost by achieving a Pt loading ratio down to 0.6–0.8 mg cm^−2^ in their innovated Pt/H_2_ systems^17^. In 2017, the U.S. Department of Energy has set a goal for further reducing the total Pt group metal in Pt/H_2_ industries down to 0.125 mg cm^−2^^18,19^.

The so-called top-coating (plating) method has long been the cornerstone in preparing Pt based electrocatalysts (electrodes) and is used to achieve high-performance/low-cost goals^20,21^. A thin Pt layer is established over the top (the surface) of a suitable substance (commonly, a metal plate), where the top-coated Pt functions as the electrocatalyst, while its ultimate weight (cost) is largely reduced. However, achieving the goal of 0.125 mg cm^−2^ in industry still remains a large challenge.

In this study, we report a novel approach to establishing high-performance/low-cost Pt-based electrocatalytic electrodes. Nanosized Pt particles are electrochemically immobilized in a monolayered manner on both surfaces of a titanium-based plate; the Pt loading ratio is reduced down to 0.0215 mg cm^−2^ while the electrocatalytic efficiency remains excellent.

## Results and Discussion

**Figure 1:**
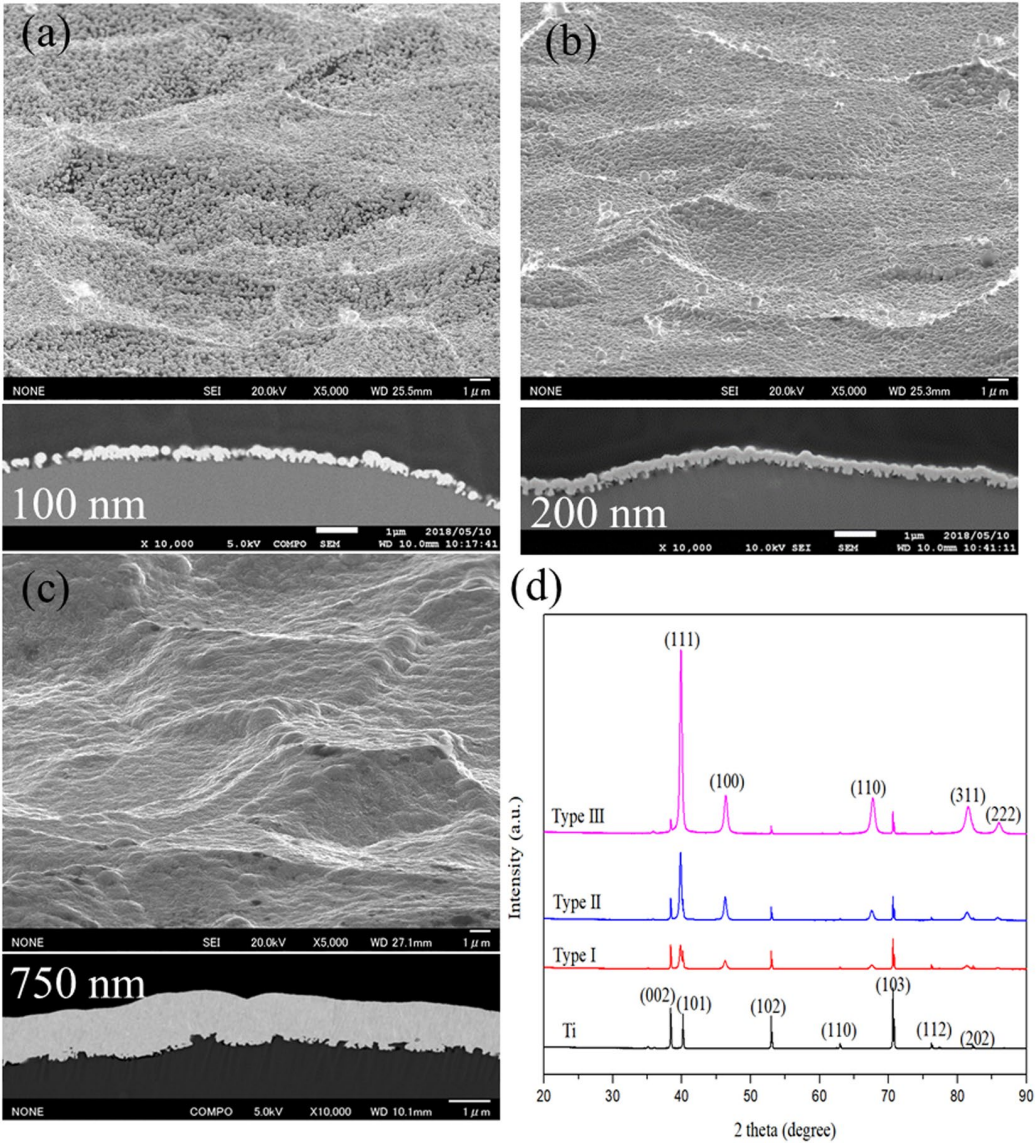
Morphological observation of the three types of Pt/Ti electrodes (Ti-plate top-coated with nanosized Pt), denoted as Type I (**a**), Type II (**b**) and Type III (**c**), as observed by SEM at an angle of 70 degrees. The underlying images of each SEM observations are the corresponding cross-sectional microstructures, showing the layered structure. The XRD patterns obtained for each type of Pt top-coated Pt/Ti electrode and the pure Ti substrate are shown in (**d**).

Three types of the Pt/Ti electrodes (Ti-plate top-coated with nano-sized Pt), denoted as Type I, Type II and Type III, are prepared; Fig. 1 shows typical SEM images, the thickness of the top-coated Pt layer on the Ti-based plate is found to be approximately 100 nm for type I (Fig. 1a), 200 nm for type II (Fig. 1b) and 750 nm for type III (Fig. 1c). The average diameter of the Pt nanoparticles is approximately 100 nm (Supplementary Information, Figure S1), Type I is nearly a monolayered type, Type II is a few-layered type, and Type III is a multilayered Pt top-coated electrode. Figure 1d shows the X-ray diffraction (XRD) patterns for each type of the Pt top-coated Pt/Ti electrodes. Characteristic diffraction peaks of face-centered cubic (fcc) Pt, which can be indexed to the (111), (100) and (110) planes (JCPDS No. 87–0647), are observed. EDX elemental analysis was performed for the Type I Pt/Ti electrode in different areas, showing the pure composition of Pt and Ti for the top-coated electrode layer and the substrate layer, respectively (Supplementary Information, Figure S2).

**Figure 2:**
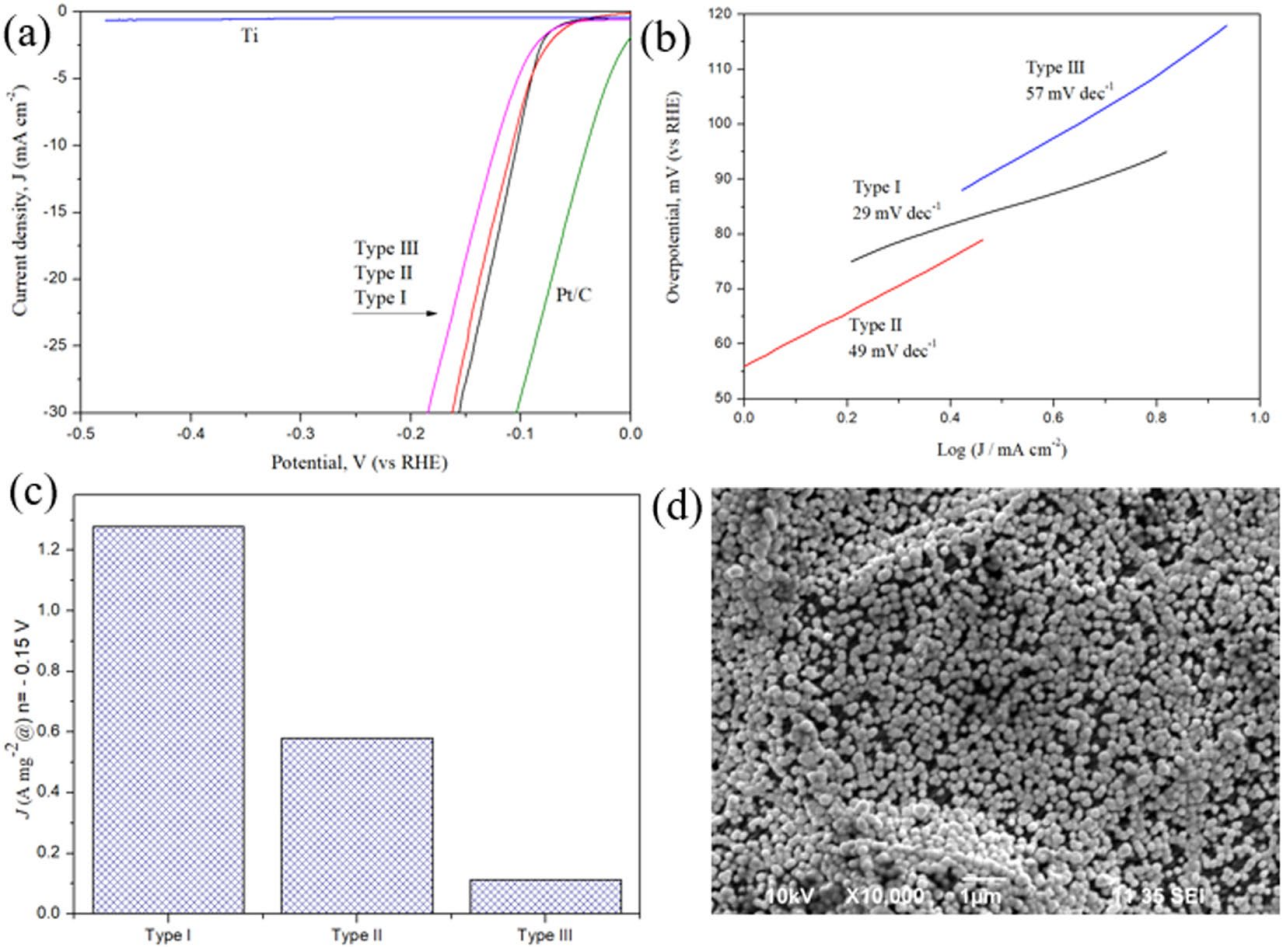
The HER polarization curves obtained for the Type I, Type II and Type III Pt top-coated Pt/Ti electrodes and the pure Ti substrate, as acquired by linear sweep voltammetry performed with a scan rate of 5 mVs^−1^ in 0.5 M H_2_SO_4_ at room temperature (**a**). The linear Tafel plots of the three types of Pt/Ti electrodes (**b**). Mass activities of the Pt/Ti electrodes at 0.15 V (versus RHE) (**c**). Uniformity of the nanosized Pt particles being coated (immobilized) on the surfaces of the Ti substance (**d**).

The electrocatalytic activities of the Pt top-coated Pt/Ti electrodes are evaluated via the electrolysis of acidic water and tap water. The activities of the pure Ti electrodes and a commercially available Pt/C electrode (the state-of-the-art electrocatalytic electrode) are also examined for comparison. Figure 2 shows the typical experimental data obtained by linear sweep voltammetry (LSV) measurements performed in 0.5 M H_2_SO_4_ at room temperature. Figure 2a shows the HER polarization curves obtained for all the Pt top-coated Pt/Ti electrodes, namely, Type I, Type II and Type III, which show excellent electrocatalytic activities toward the HER. Among the Type I, Type II and Type III electrodes, Type I showed the highest electrocatalytic activity, followed by Type II and then Type III. An increase in the thickness of the top-coated Pt layer resulted in a decrease in the electrocatalytic activity of the Pt top-coated Pt/Ti electrodes. The detrimental effect of the thickness of the top-coated Pt layer was also observed in previous studies^21^. The linear Tafel plots (Fig. 2b) are fit well by the Tafel equation (η = b log j + a, where j is the current density, and b is the Tafel slope). The following three steps (reactions) are the essential reactions involved in the hydrogen evolution reaction under acidic conditions on the metal electrode surfaces via the electrocatalyst^22^:

1 $${\rm{Volmer}}\,{\rm{step}}:{{\rm{H}}}_{3}{{\rm{O}}}^{+}+{{\rm{e}}}^{-}\rightleftarrows {{\rm{H}}}_{2}{\rm{O}}+{\rm{H}}({\rm{ad}})\,({\rm{Tafel}}\,{\rm{slope}}\,120\,{\rm{mV}}\,{{\rm{dec}}}^{-1})$$

2 $${\rm{T}}{\rm{a}}{\rm{f}}{\rm{e}}{\rm{l}}\,{\rm{s}}{\rm{t}}{\rm{e}}{\rm{p}}:\,2{\rm{H}}({\rm{a}}{\rm{d}})\rightleftarrows {{\rm{H}}}_{2}({\rm{g}})\,({\rm{T}}{\rm{a}}{\rm{f}}{\rm{e}}{\rm{l}}{\rm{s}}{\rm{l}}{\rm{o}}{\rm{p}}{\rm{e}}\,30\,{\rm{m}}{\rm{V}}\,{{\rm{d}}{\rm{e}}{\rm{c}}}^{-1})$$

3 $${\rm{Heyrovsky}}\,{\rm{step}}:{\rm{H}}({\rm{ad}})+{{\rm{H}}}_{3}{{\rm{O}}}^{+}+{{\rm{e}}}^{-}\to {{\rm{H}}}_{2}({\rm{g}})+{{\rm{H}}}_{2}{\rm{O}}({\rm{Tafel}}\,{\rm{slope}}\,40\,{\rm{mV}}\,{{\rm{dec}}}^{-1})$$

where e^-^ denotes metal-bound electrons, and H(ad) and H_2_(g) represent a hydrogen atom and a hydrogen molecule being adsorbed on the surface of a metal atom, respectively. The Tafel slopes are estimated to be 29 mV dec^−1^, 49 mV dec^−1^ and 57 mV dec^−1^ for the Type I, Type II and Type III Pt/Ti electrodes, respectively, suggesting that the Tafel step is most likely to be the rate-determining step for the Type I electrode; meanwhile, for the Type II electrode and Type III electrode, the Heyrovsky step is the rate-determining step. The Type I Pt/Ti electrode exhibits a Tafel slope of 29 mV dec^−1^ and is comparable or even better than many recently reported metal-/carbon-based HER catalysts (the detailed comparison is listed in Supplementary Table S1). The state-of–the-art Pt/C electrode shows a Tafel slope of 31 mV dec^−1^, which is in consistent with the value reported in previous studies^23^.

The specific electrocatalytic activity of each electrode was calculated from the polarization curves by normalizing the current with the geometric area of the electrode. The specific electrocatalytic activity is found to be 27.5 mA cm^−2^ for Type I, 24.9 mA cm^−2^ for Type II, and 18.2 mA cm^−2^ for Type III (Supplementary Figure S3) at an overpotential of 0.15 V. By normalizing based on the Pt loading mass (Fig. 2c), the electrocatalytic activity was found to be 1.28 A mg^−1^ for the Type I electrode at an overpotential of 0.15 V. The electrocatalytic activity of the Type I electrode is 2.1 times higher than that of the Type II Pt/Ti electrode (0.58 A mg^−1^) and is approximately 11.3 times greater than that of the Type III Pt/Ti electrode (0.11 A mg^−1^). The Type I electrode showed the best cost/performance among all the types of the Pt/Ti electrodes. A video is recorded to demonstrate the visible electrocatalytic activity of the Type I electrode during the experiment. Plenty of H_2_ bubbles were steadily coming out from the electrode surface (video file). Figure 2d shows the uniformity of the nanosized Pt particle that was firmly immobilized on the surfaces of the Ti substrate.

The charge transfer resistance (R_ct_), which reflects the electrocatalytic kinetics^24^, is also calculated (Supplementary Figure S4) and found to be 2.0 ohms for Type I, 2.3 ohms for Type II and 2.4 ohms for Type III.

**Figure 3:**
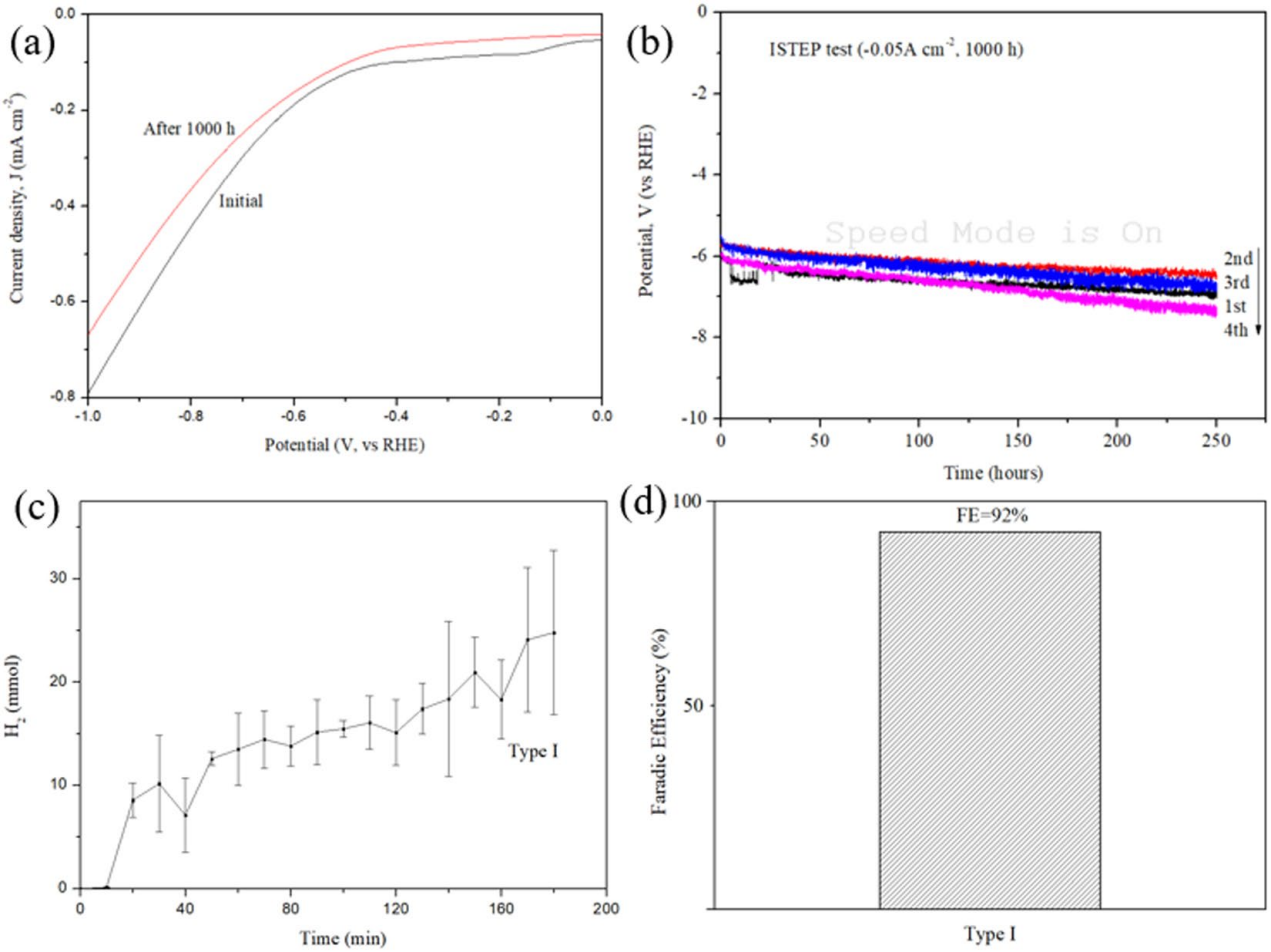
Long-term (1000 hours of the electrolysis of tap water) stability measurements performed with the Type I Pt/Ti electrodes for the electrolysis of tap water. The linear sweep voltammetry (LSV) curves were recorded using a scan rate of 5 mVs^−1^, initially and after 1000 hours of the ISTEP measurements (**a**). ISTEP measurements obtained during the 1000-hour EHW electrolysis process performed at a current density of 0.05 A cm^−2^ in tap water, and four continuous cycling tests of 1^st^, 2^nd^, 3^rd^, 4^th^ ISTEP experiment are noted (**b**). The resultant amount of H_2_, denoted in mmol, obtained by the electrolysis of tap water, as quantified by TRIlyzer mBA-3000 system (**c**), and the HER Faradic efficiency (FE) of the Type I Pt/Ti electrode (**d**).

Making electrolyzed hydrogen water (EHW) from real tap water via electrocatalytic interactions remains a large challenge, even today. It is well known that under a higher pH (cathode, pH > 10), Mg^2+^ and Ca^2+^ ions, the two common divalent cations involved in tap water, tend to form insoluble hydroxides on the electrode surfaces^25^. This difficulty is also encountered for Pt/Ti electrodes in the long-term electrolysis of tap water. After the long-term electrolysis of tap water, the electrode surface is found to be partially covered with precipitates due to the divalent cations in the tap water, which is confirmed by XPS (Supplementary Figure S5). Figure 3 summarizes the experimental data obtained for the long-term (1000 hours of the electrolysis of tap water) stability studies performed at a constant current density of 0.05 A cm^−2^. The linear sweep voltammetry (LSV) curves obtained initially and after 1000 hours of the ISTEP measurements are shown in Fig. 3a. Figure 3b shows the ISTEP measurements obtained during the 1000 hour EHW electrolysis process (4 cycles, each performed for 250 hours) performed at a current density of 0.05 A cm^−2^ in tap water. The overpotential increased constantly with a ratio of 3.2 mV h^−1^ during the 1000 hours of tap water electrolysis; this in turn is in an increase in energy consumption, which is denoted as kWh per kg of H_2_. The energy consumption for the electrolysis of 1 kg H_2_ is calculated to be 85.7 kWh (the calculation method is given in Supplementary Table S2). The HER polarization curves and the corresponding electrochemical impedance spectra observed for the electrolysis of tap water with a pH of 6.8 are shown in Supplementary Figure S6. The HER polarization curve obtained in a sea water with a pH of 8.0 (the Tafel slope is estimated to be 198 mV dec^−1^) for the Type I Pt top-coated Pt/Ti electrode is shown in Supplementary Figure S7. The amount of H_2_ produced via the electrolysis of tap water was quantified by an H_2_ analyzer. Abundant H_2_ and O_2_ bubbles were observed on the electrode surface, and they dissipated quickly into the tap water. The results of the mole number of H_2_ are shown in Fig. 3c. The Type I Pt/Ti electrode gave an HER Faradaic efficiency (FE) of 92% **(**Fig. 3d**)**^26^. A calculated 0.14 mmol of H_2_ per minute can be produced by the electrolysis of real tap water via the Type I Pt/Ti electrode.

**Figure 4:**
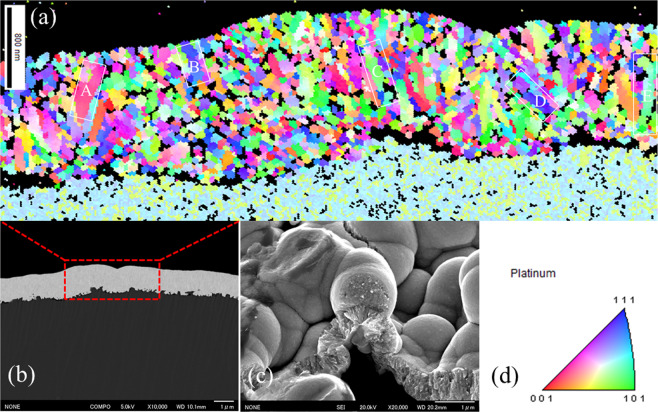
Cross-sectional electron backscatter diffraction (EBSD) image of the Type III Pt/Ti electrode, where the main grains grow vertically toward the Pt-Ti interface and are labeled A, B, C, D and E (**a**). Cross-sectional SEM images of the Type III Pt/Ti electrode (**b, c**). In the color legend, the three corners of the triangle represent the three basal planes of Pt, the (111), (001) and (101) facets, along the cross-sectional direction (**d**).

The cross-sectional microstructures of the Pt-top-coated Pt/Ti electrodes were evaluated by electron backscatter diffraction (EBSD). Figure 4a shows an inverse pole plot of the Type III Pt/Ti electrode. In the color legend, the three corners of the triangle represent the three basal planes of Pt. The facets (111), (001) and (101), which are located on the side-lines, are vicinal (or stepped) planes, while the inside of the triangle represents high-index (or kinked) planes^27^. The EBSD images indicate that all the Pt catalyst grains in the scanned area are high-index orientations of distinctly different structures. Comparing the cross-sectional structures observed in the SEM images (Figure b and c) with the EBSD results, four main grains can be identified, which are denoted as green, yellow, red and blue. In addition, the main grains grow vertically toward the Pt-Ti interface, which are labeled A, B, C, D and E. The inverse pole figure (IPF) maps obtained along the [010] direction of the scanning area display that the crystallographic direction located at the (111) facet represents the highest texture density (Supplementary Figure S8). Other domains at different locations have also been identified in the same way, as shown in Supplementary Figure S9.

**Figure 5:**
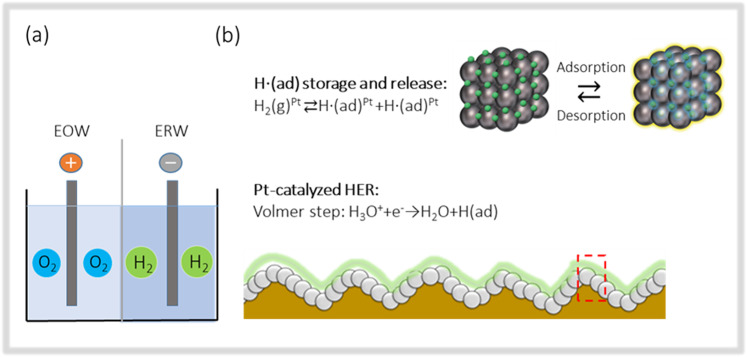
Production of EHW via the Pt-top-coated Pt/Ti electrodes. The EHW near the cathode is hydrogen-rich water, whereas the EHW near the anode contains oxygen (**a**). Chemical reactions on the surface of the Pt/Ti electrodes. The solvated protons (H_3_O^+^) in the electrolyte have been transported into the water and the hydrogen atoms and/or molecules. The hydrogen molecules have been further transformed into the reactive hydrogen over the Pt nanoparticles. The reactive hydrogen species penetrate into the Pt clusters, and the desorption takes place simultaneously (**b**).

A model, as shown in Fig. 5, is proposed to illustrate the key characteristics of EHW produced via the Pt-top-coated Pt/Ti electrodes based the electrocatalytic interactions. The key concepts of this model are summarized as follows: i) the three essential species of water, i.e., water molecules (H_2_O), hydronium ions (H_3_O^+^), and hydroxyl ions (OH^−^), are adsorbed properly on the Pt (111) facet^28^; ii) on the cathode, hydronium ions receive electrons and decay into hydrogen (H*) and water (H_2_O), namely, by the Volmer step; iii) H* is highly reactive but can be stabilized either via penetration into Pt or the formation of hydrogen molecules (H_2_)^29^; iv) the reactive H* species that have penetrated into Pt can be stored for a long period of time^30,31^ v) a certain amount of the nanosized Pt particles in which H* species are stored escaped to the EHW (EHW was reported to contain 12 ppb Pt nanoparticles^14^, and the amount of Pt in the ERW increased along with the intensity of electrolysis^32^); vi) EHW retains part of the biological activity, i.e., the anti-cardio-renal injury effect^33^ and scavenging activity of reactive oxygen species in cells^32^, even after de-H_2_ gas treatment, in contrast to the hydrogen water produced by bubbling hydrogen gases; vii) the distinguishing properties, namely, the healthy beneficial effects of EHW, are attributed to the highly reductive properties of the H*-Pt nanoparticles in the EHW.

It is noted here that titanium (Ti) is a desirable underlying substance for the creation of the Pt-top-coated electrocatalytic electrodes. Strong metal-support interactions (SMSIs) exist between the Pt atoms and the Ti substrate, which has been demonstrated to occur preferentially in the case of noble metals highly dispersed with small Pt particles^34–37^. The use of a Ti substrate with a large surface area (porous structure, as seen in the cross-sectional EBSD image) offers a strong metal-support interaction, which plays a crucial role in enhancing the activity and the stability of our Pt-top-coated Pt/Ti electrodes. The so-called density functional theory (DFT) is used to quantitatively evaluate the effect of the substrate-induced interfacial discrepancy on the electronic structure of the Pt/Ti electrodes. A useful descriptor of these changes in the electronic structure is the hydrogen binding energy (HBE), a parameter that depends strongly on the HER activity. Table 1 summarizes the HBE on the Pt (111), Ti (111), and Pt (111) surfaces of the Type I Pt/Ti electrode. Ti enhanced the adsorption ability of Pt for hydrogen atoms, thus finally facilitating the process of the HER.

**Table 1:** ^*^DFT-calculated hydrogen binding energies (HBEs) estimated based on DFT for the Ti, Pt, and Pt of the Type I Pt-top-coated Pt/Ti surfaces.

Surface	HBE [eV]
Ti (111)	−1.23
Pt (111)	−0.49
Type I Pt/Ti	−0.52

^*^See the Experimental Section for more details on the HBE calculations.

The top-coated Pt/Ti electrodes were scaled up to 7.5 × 11.5 cm and were used for mass production of EHW. Inductively Coupled Plasma/Mass Spectrometry (ICP-MS) was used for quantitating the amount of Pt nanoparticles detached from the Pt/Ti electrode in EHW; Table 2 summarizes the analytical data. The as-produced EHW was directly analyzed and 3.3 ± 0.2 ppb (n = 3) Pt was detected. A small amount of concentrated HCl was spiked to EHW (concentration of HCl in the final EHW sample is approximately 5.0%), the sample was then directly analyzed and 5.1 ± 0.1 ppb (n = 3) Pt was detected. The as-produced EHW was filtrated through a 20 nm pored alumina filter and the filtrate was detected; 1.1 ± 0.1 ppb (n = 3) Pt was detected. Tap water was used for making the EHW and no Pt was detected. A small amount (0.15 ppb, average of 3 measurements) of Pt was found also in the oxygen-contained electrolyzed water.

**Table 2:** Quantitative detection of Pt in electrolyzed hydrogen water (EHW) samples (n = 3) via ICP-MS (Parkin Elmer Elan DRC-e). The calibration curve was obtained by using the standard samples of 15, 7.5, 5.0, 2.0, and 1.0 ppb Pt contained 5.0% HCl. The 20 nm pored alumina filter was obtained from Whatman.

Samples	Tap water	EHW	EHW + 5.0% HCl	Filtered EHW
Pt (ppb)	ND	3.3 ± 0.2	5.1 ± 0.1	1.1 ± 0.1

^*^ND: Not detectable.

Supply of Pt nanoparticles into EHW in a constant manner is the key to store the reactive hydrogen (·H*) with a longer time. Life span which means the electrocatalytic efficiency decayed to 70% of the initial efficiency of the 7.5 × 11.5 cm sized top-coated Pt/Ti electrodes is approximately 3500 kg-EHW per electrode.

Water is the most important and abundant element to life. Drinking of the nano-Pt contained EHW shall be a direct solution to promote healthier life. The pH for EHW for drinking is restricted in the range of pH 9.5 ± 0.3. Our top-coated Pt/Ti electro-catalytic electrodes are recommended solely for use in EHW production.

**Figure 6:**
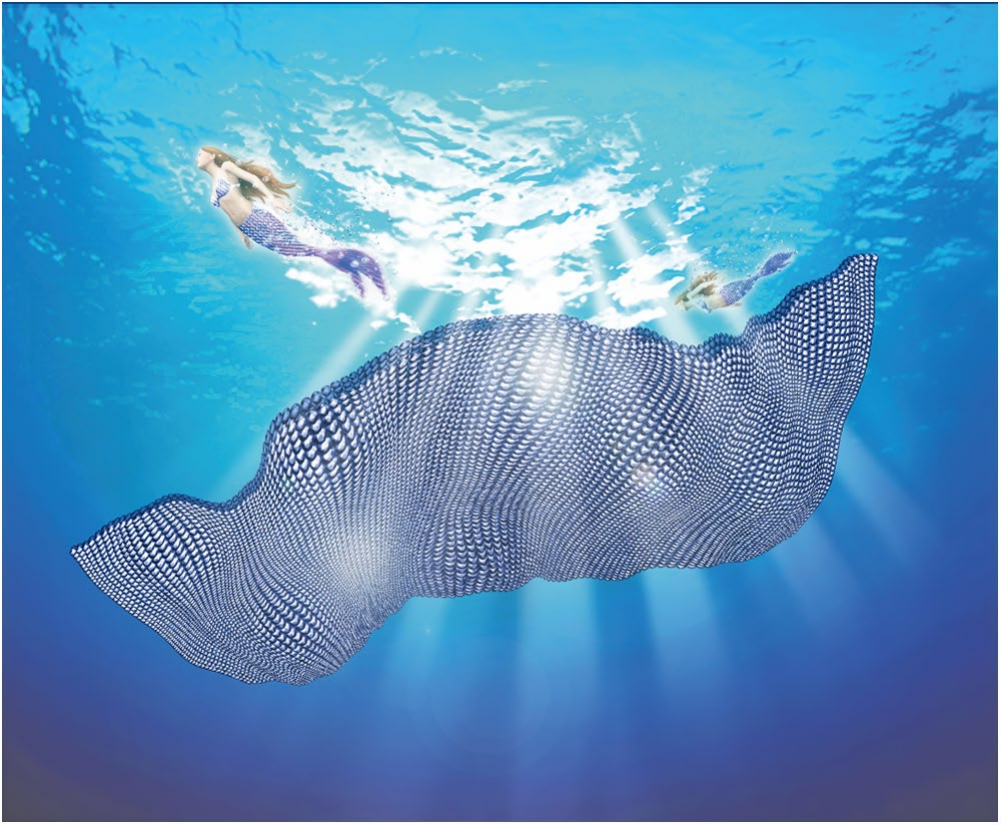
An illustration (created by a scientific illustrator, Shinichiro Kinoshita) showing the effects of Pt-H* on health benefits. A dreamful mermaid is used to illustrate the springtime of life; the mermaid has been fully recharged by drinking the Pt-H*-containing EHW. The wavy metal plate is used to show the monolayered platinum nanoparticles, which are the key for generating and storing H* species. The ocean is used to represent the eco-environments of all life.

In summary, we have demonstrated experimentally that the loading ratio of Pt can be reduced down to 0.0215 mg cm^−2^ while maintaining excellent electrocatalytic performances. Top-coating Pt nanoparticles on the surfaces of Ti plates is the key technology for achieving this goal. EHW produced via the Pt top-coated Pt/Ti electrodes contains certain numbers (approximately 12 ppb) of free Pt nanoparticles. The highly reductive hydrogen (H*) species penetrate into Pt nanoparticles, where they are stored for a long period of time. The biological features of EHW, especially the distinguishing reductive properties, can be attributed to the intrinsic properties of Pt-H*. Hydrogen is the first and the lightest element in the periodic table. Hydrogen atoms, even hydrogen molecules, are small enough to penetrate and/or pass through most naturally occurring or even human-made substances. Hydrogen, once it is activated, i.e., the H* species obtained via the hydrogen/platinum catalytic interaction, is a particularly important species. H* can penetrate into any part of our body and is capable of selectively reacting with reactive oxygen species (ROS). An illustration showing the potential health benefits of H* is given in Fig. 6: a dreamful mermaid is fully recharged and is, right now, going to explore the fascinating new world by drinking the Pt-H*-containing EHW.

## Experimental Section

### Preparation of Pt/Ti electrodes

The Ti plate, used as the cathode, and Pt/Ti plate, used as the anode, are immersed in a platinum plating solution and connected to a power supply used for energizing to prepare various types of the Pt-coated Pt/Ti electrodes. Three types of the Pt-coated Pt/Ti electrodes (Ti-plate top-coated with nanosized Pt), denoted as Type I, Type II and Type III, are prepared. Thickness of the top-coated Pt layer is approximately 100 nm, 200 nm, and 750 nm for Types I, II and III, respectively. A ratio of 1.0 μm/30 min is optimized and is used to prepare all the Pt-coated Pt/Ti electrodes throughout this study. Nihon Trim Co. Ltd. is willing to donate the Pt-coated Pt/Ti electrodes, but the donation is surely subject to the academic studies on EHW.

### Preparation of Pt/C electrodes

Glassy carbon electrodes (GCEs) (inner diameter (ID): 3 mm, outer diameter (OD): 6 mm, area: 0.07 cm^2^) obtained from CHI Instruments were used. The GCEs were polished with polishing diamond (1.0 μm from CHI Instruments) suspended in distilled water on a diamond polishing pad and alumina powder (0.05 μm from CHI Instruments) suspended in distilled water on an alumina polishing pad (CHI instruments). After the first and second stages of cleaning, the electrodes were thoroughly rinsed with deionized water. Before being loaded with Pt, the electrodes were also cleaned by immersing them in an isopropanol solution and sonicating for approximately 10 s. The electrodes were dried overnight at ambient conditions for further use. Ag/AgCl (with 3 M NaCl as the filling solution) and platinum foil were used as the reference and counter electrode, respectively. Typically, 4 mg of the Pt nanosized powders were dispersed in a 600 μl mixture of water and ethanol (17:13, v/v), and then, 400 μl of a Nafion solution (0.5 wt % in water) was added. This suspension was immersed in an ultrasonic bath for 30 min to prepare a homogeneous ink (4 mg ml^−1^). The Pt/C electrode was prepared by depositing 12.5 μl of the Pt-containing ink onto the GCE (the Pt loading ratio is 0.14 mg cm^−2^, and the ultimate Pt loading value is 0.01 mg for each Pt/C electrode).

### Electrochemical measurements

The HER activity of various Pt-coated Pt/Ti electrodes (with sizes of 1 cm ×0.5 cm) was tested in 0.5 M H_2_SO_4_ (pH = 0.18), tap water (Tokyo, pH = 6.8) and sea water (Izu seawater, pH = 8.0) at room temperature using a standard three electrode setup on an electrochemical workstation (CHI608C, CHI Instrument). Tap water was purified by TRIM TI-5HX (NIHON TRIM) before use. During the analysis, the Pt-coated Pt/Ti electrode was clamped by a customized crocodile-clip-electrode with sizes of 0.5 cm  × 0.5 cm immersed in the electrolyte solution.

To condition the electrodes, 50 CV cycles were conducted between 0.0 V (vs. the normal hydrogen electrode, RHE) and 0.5 V at 100 mVs^−1^. Tafel curves were then obtained by performing linear sweep voltammetry using a scan rate of 5 mVs^−1^. EIS measurements were conducted in a static solution at an initial potential of −0.26 V (vs. Ag/AgCl). The amplitude of the sinusoidal wave was 5 mV, and the frequency scan range was from 100 kHz to 0.1 Hz. Each of the samples was measured at least three independent times, and the average is used. Unless otherwise stated, all the experiments were performed at ambient temperature (23 ± 2 °C), and the electrode potentials were converted to the RHE scale using E(RHE) = E(Ag/AgCl) + 0.197 V + 0.059*pH.

### ICP-MS analysis of EHW

Quantitative detection of Pt in electrolyzed hydrogen water (EHW) samples (n = 3) via ICP-MS (Parkin Elmer Elan DRC-e) were analyzed for elements present in the samples. TW refers to tap water, EHW + 5% HCl refers to that the concentration of HCl in the final EHW sample is approximately 5%, and Filtered EHW refers to the filtrate of EHW, which was purified by filtration.

### Detection of H_2_

The amount of H_2_ gas generated by a sealed electrocatalytic cell was measured by using a TRIlyzer system (mBA-3000, TAIYO Instrument). The Faradaic yield was calculated from the total charge *Q*(C) that passed through the cell and the total amount of hydrogen produced *n*_*H*2_ (mol). *Q* = t/1,000 (C), where t is the time (s) under a constant reduction current. Assuming that two electrons are needed to produce one H_2_ molecule, the Faradaic efficiency can be calculated as follows^26^:

$$\eta =\frac{4F\ast {n}_{{H}_{2}}}{Q}$$

where *F* is the Faraday constant, and *n*_*H2*_ is the number of moles of H_2_.

### Computational method

Our calculations are performed based on DFT calculations, as implemented in the Vienna ab initio package^38,39^. The general gradient approximation of Perdew–Burke–Ernzerhof is adopted for the exchange-correlation functional^40^. Moreover, the electron wave functions were expanded by a plane wave cutoff of 550 eV. The (4 × 4 × 1) supercell containing 48 Ti atoms and 64 Pt atoms was constructed by a periodic boundary condition, and the vacuum layers were set to be larger than 20 Å to avoid periodic interactions. Reciprocal space was represented by the Monkhorst-Packspecial k-point scheme, and 4 × 4 × 1 grid meshes were used to represent the structure relaxation of the Pt/Ti system. Atomic relaxation was performed until the total energy variation was smaller than 10^−6^ and all the forces on each atom were less than 0.01 eV/Å.

## Supplementary information

Supplementary information.

Supplementary video.
