# Supplementary material for: Monolayered Platinum Nanoparticles as Efficient Electrocatalysts for the Mass Production of Electrolyzed Hydrogen Water
Source: Sci Rep. 2020 Jun 23;10:10126. doi: 10.1038/s41598-020-67107-1 (PMC7311417; doi:10.1038/s41598-020-67107-1)
Supplement: Supplementary file 1 — Supplementary information. [file 41598_2020_67107_MOESM1_ESM.docx]

**Monolayered Platinum Nanoparticles as Efficient Electrocatalysts for the Mass Production of Electrolyzed Hydrogen Water**

Yanqing Wang ^a, b*^, Bunshi Fugetsu ^c**^, Ichiro Sakata ^c, d^, Chika Fujisue ^d^, Shigeru Kabayama ^b^, Norio Tahara ^b^, Shinkatsu Morisawa ^b^

^a^ College of Polymer Science and Engineering, Sichuan University, Chengdu 610065, China

^b^ Nihon Trim Co. Ltd, Oyodonaka, Kita-ku, Osaka, Japan

^c^ Institute for Future Initiatives, The University of Tokyo, Bunkyo-ku, Tokyo, 113-0032, Japan

^d^ School of Engineering, The University of Tokyo, Bunkyo-ku, Tokyo, 113-0032, Japan

* Corresponding author.

** Corresponding author.

E-mail: yanqingwang@scu.edu.cn (Y. Wang), bunshifugetsu@ifi.utokyo.ac.jp (B. Fugetsu).

**Supplementary Information**


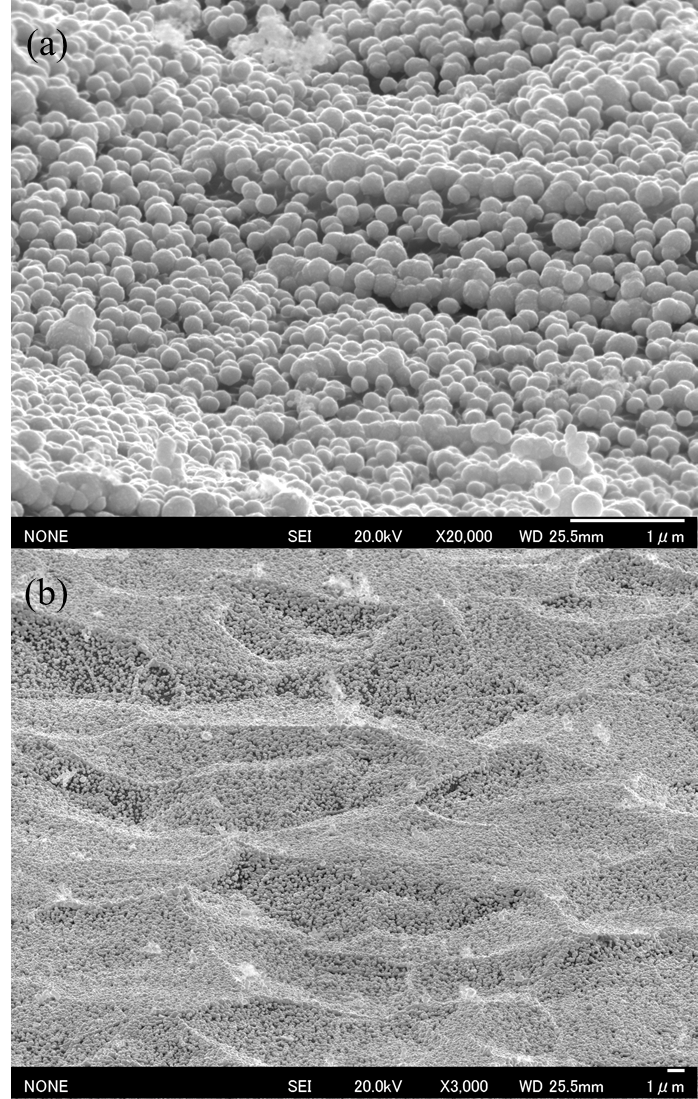


Figure S1. Detailed morphological structure of monolayered Pt top-coated Pt/Ti electrode (Type I) at an angle of 70 degrees.


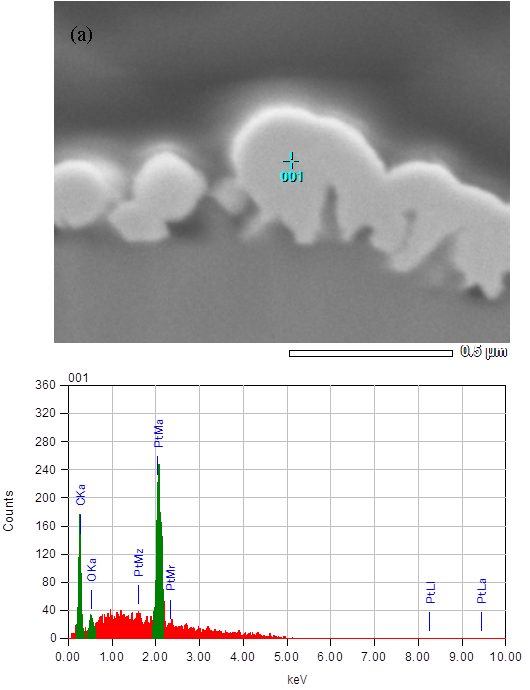


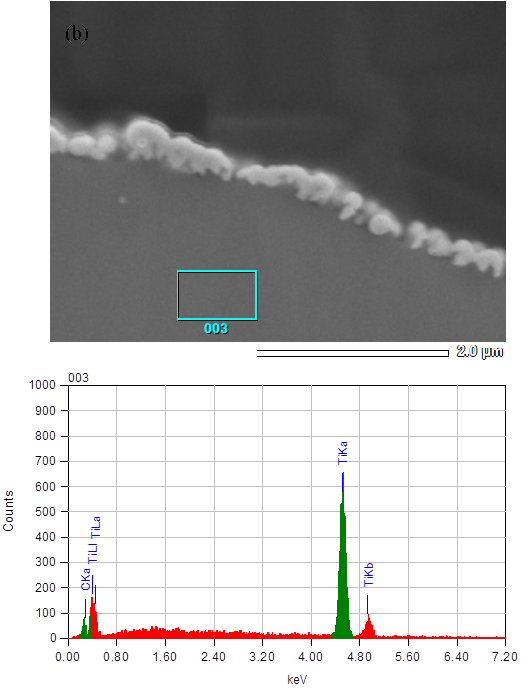


Figure S2. EDX elemental analysis obtained for Type I Pt/Ti electrode, with the top-coated electrode layer (a) and substrate layer (b).

Table S1. Comparison of the HER activity of recently reported metal-/carbon-based electrocatalysts.

| Catalyst | Support | Loading  (mg cm^-2^) | Tafel slope (mV dec^-1^) | η_10_  (mV) | Ref. |
| --- | --- | --- | --- | --- | --- |
| Pt | Ti | 0.0215 | 29 | 103 | *This work* |
| Ni/Ni(OH)_2_ | graphite | ― | 116 | ~120 | *Proceedings of the National Academy of Sciences* ***2017,*** *114 (34), 8986* |
| Pt cuboid/rGO | glassy carbon | 0.170 | 29 | ~75 | *Journal of Power Sources* ***2015,*** *285, 393* |
| MoNi_4_/MoO_2_ | Ni | ~43.4 | 30 | ~15 | *Nature Communications* ***2017,*** *8, 15437* |
| NiO/Ni-CNT | RDE | 0.28 | 82 | ~80 | *Nature Communications* ***2014,*** *5 (1), 4695* |
| MoS_2(1−_*_x_*_)_Se_2_*_x_* | NiSe_2_ | 4.5 | 42.1 | 69 | *Nature Communications* ***2016,*** *7 (1), 12765* |
| Fe_1–_*_x_*Co*_x_*S_2_/CNT | RDE | 0.4 | 46 | 160 | *Journal of the American Chemical Society* ***2015,*** *137 (4), 1587* |
| Boron Nitride (h-BN) | Au | ― | 27 | 70 | *Scientific Reports* ***2016,*** *6 (1), 32217* |
| MoS_2_-PPy | RRDE | 0.28 | 80.5 | 251 | *Scientific Reports* ***2017,*** *7 (1), 42309* |
| N-CoP_2_ | glassy carbon | ― | 46 | 38 | *Science Advances* ***2020,*** *6 (1), eaaw8113* |
| Sr_2_RuO_4_ | glassy carbon | 0.464 | 51 | 61 | *Nature Communications* ***2019,*** *10 (1), 149* |
| Pt–Ru dimers | RDE | 0.00167 | 28.9 | 22 | *Nature Communications* ***2019,*** *10 (1), 4936* |
| Ru-MoS_2_ | Carbon cloth | 12.40 | 114 | 45 | *Applied Catalysis B: Environmental* ***2019,*** *249, 91* |
| Ru@C_4_N | GCE | 2.7 | 26 | 6 | *ACS Applied Materials & Interfaces* ***2019,*** *11 (21), 19176* |


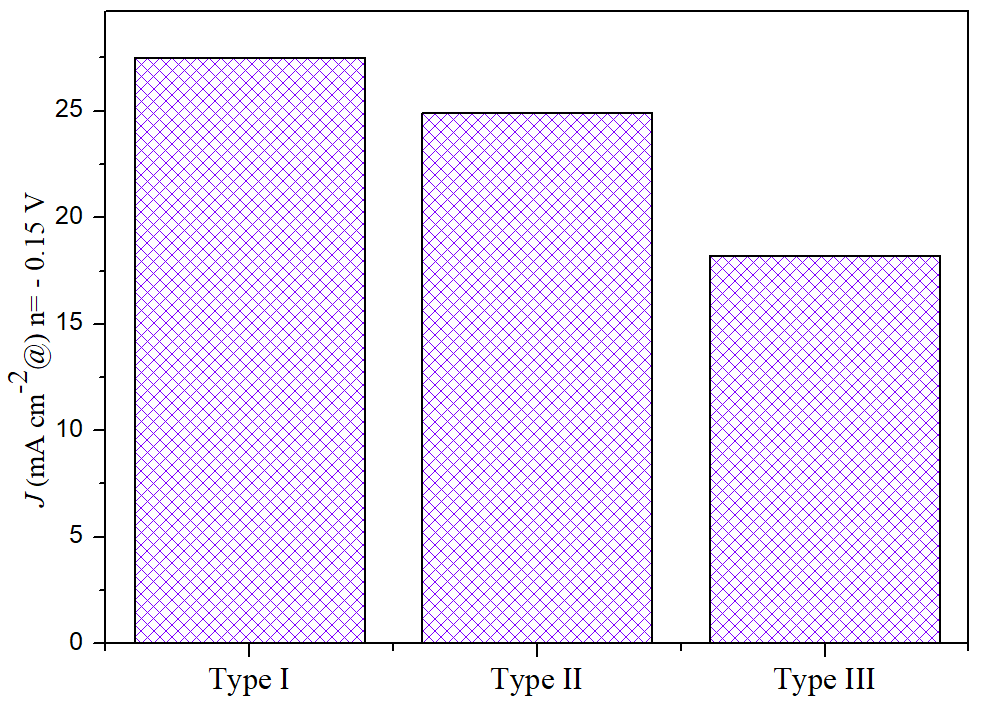


Figure S3. LSV-derived HER activity at 0.15 V (versus RHE) obtained for Type I, Type II and Type III Pt top-coated Pt/Ti electrodes performed in 0.5 M H_2_SO_4_ at room temperature.


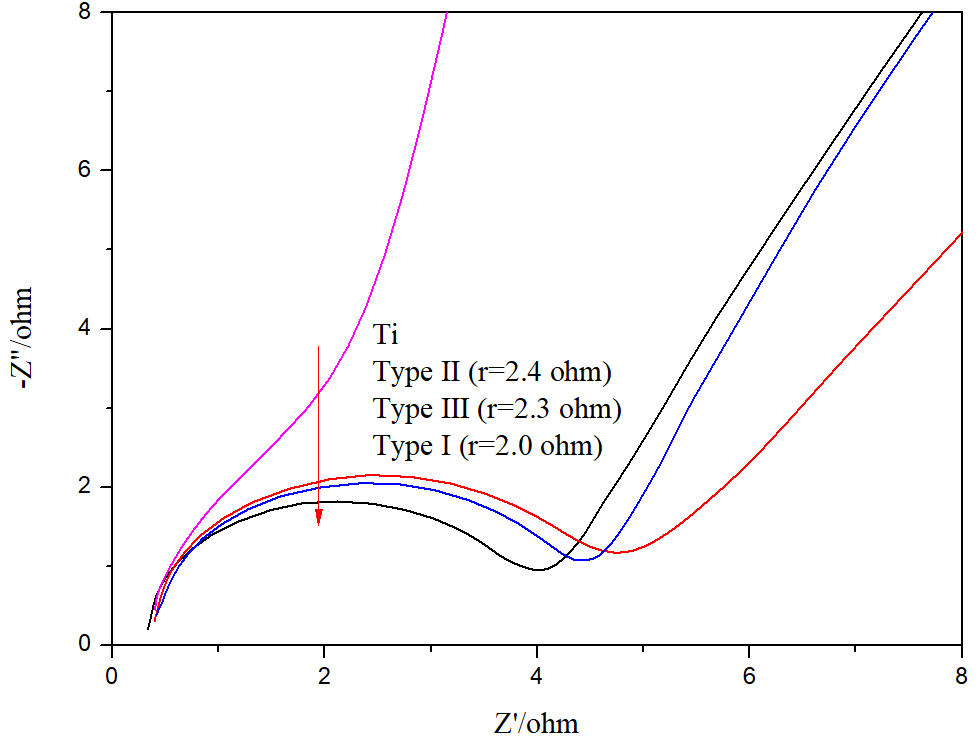


Figure S4. Electrochemical impedance spectra (EIS) obtained for Type I, Type II, Type III Pt top-coated Pt/Ti electrodes and Ti substrate performed in 0.5 M H_2_SO_4_ at room temperature.


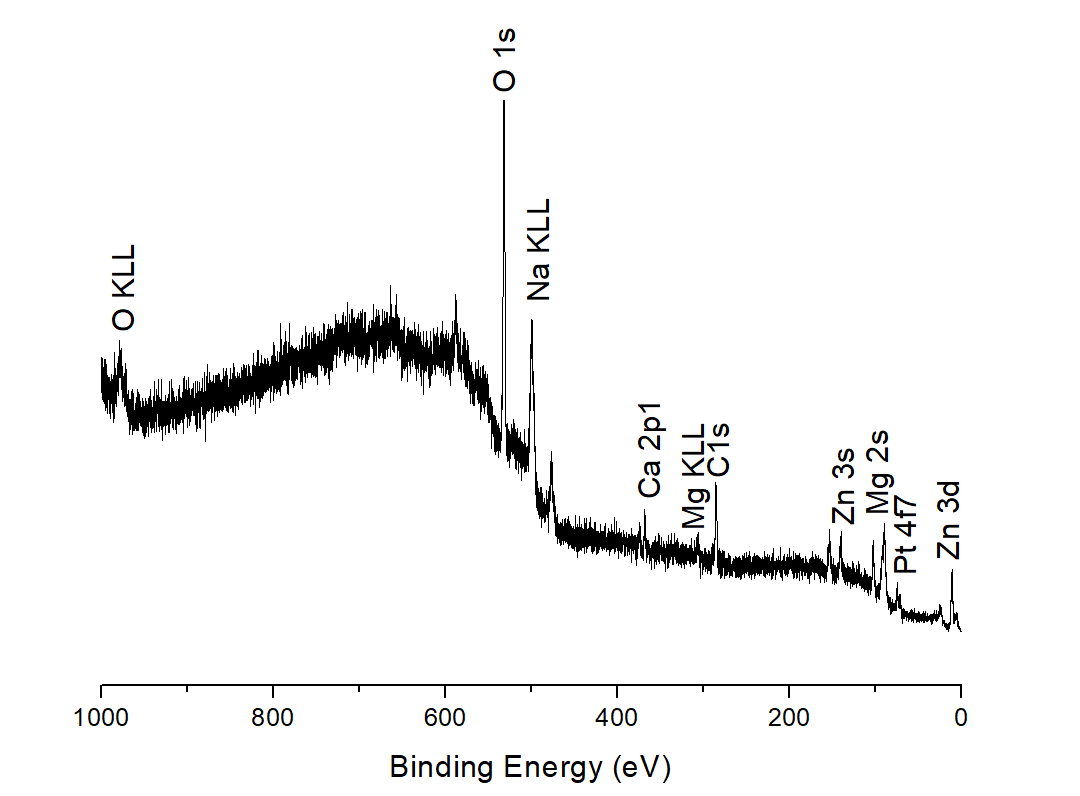


Figure S5. XPS general spectrum obtained for Type I Pt top-coated Pt/Ti electrode after 1000-hour EHW electrolysis process.


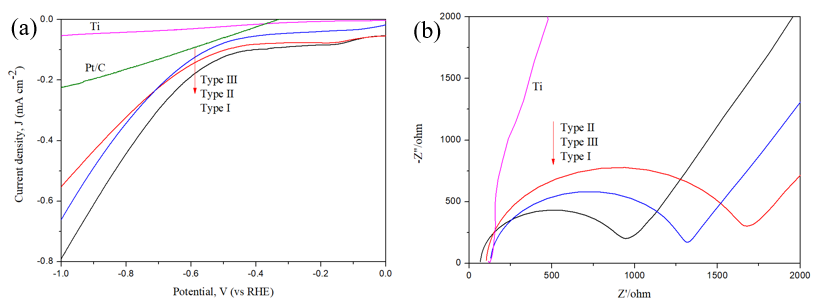


Figure S6. The HER polarization curves obtained for Type I, Type II, Type III Pt top-coated Pt/Ti electrodes, Pt/C, and Ti substrate, as acquired by linear sweep voltammetry performed with a scan rate of 5 mVs^-1^ in tap water (pH=6.8) at room temperature (a). The electrochemical impedance spectra (EIS) of Type I, Type II, Type III Pt top-coated Pt/Ti electrodes and Ti substrate (b).


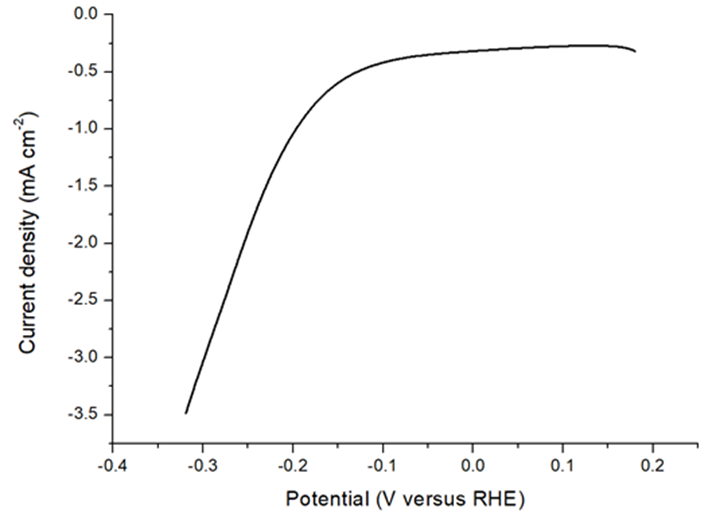


Figure S7. The HER polarization curves obtained for Type I Pt top-coated Pt/Ti electrode, as acquired by linear sweep voltammetry performed in sea water (pH=8.0) with a scan rate of 5 mVs^-1^ at room temperature.


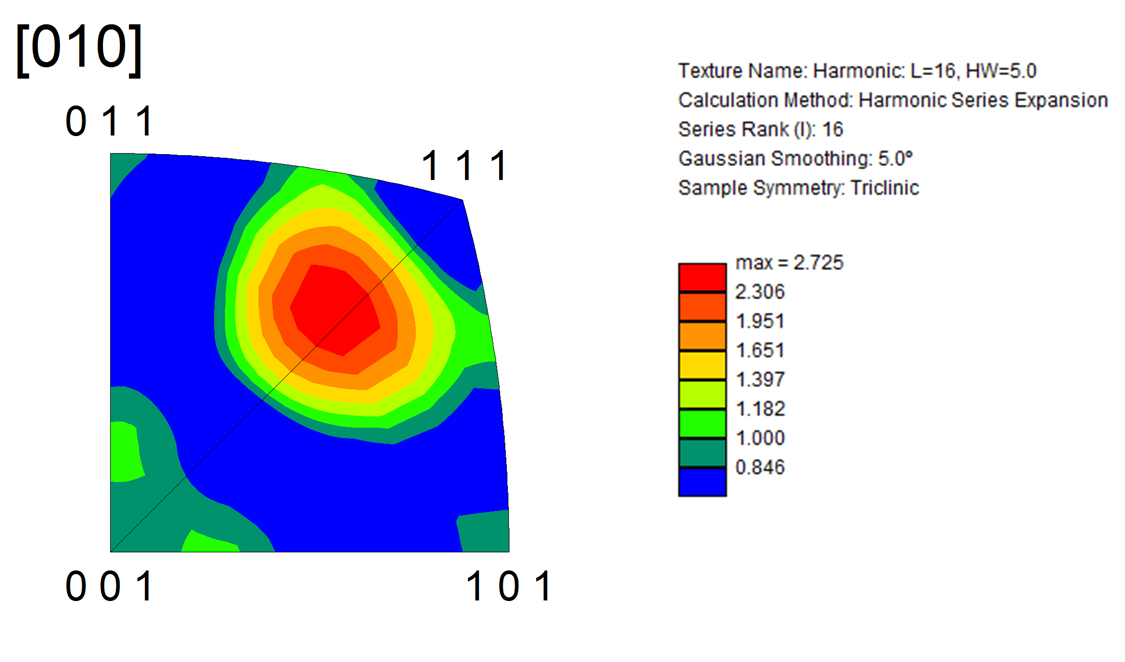


Figure S8. A fitting process by inverse pole figure (IPF) maps at [010] direction of the scanning area displays that the crystallographic direction located at facet (111) represents the highest texture density.


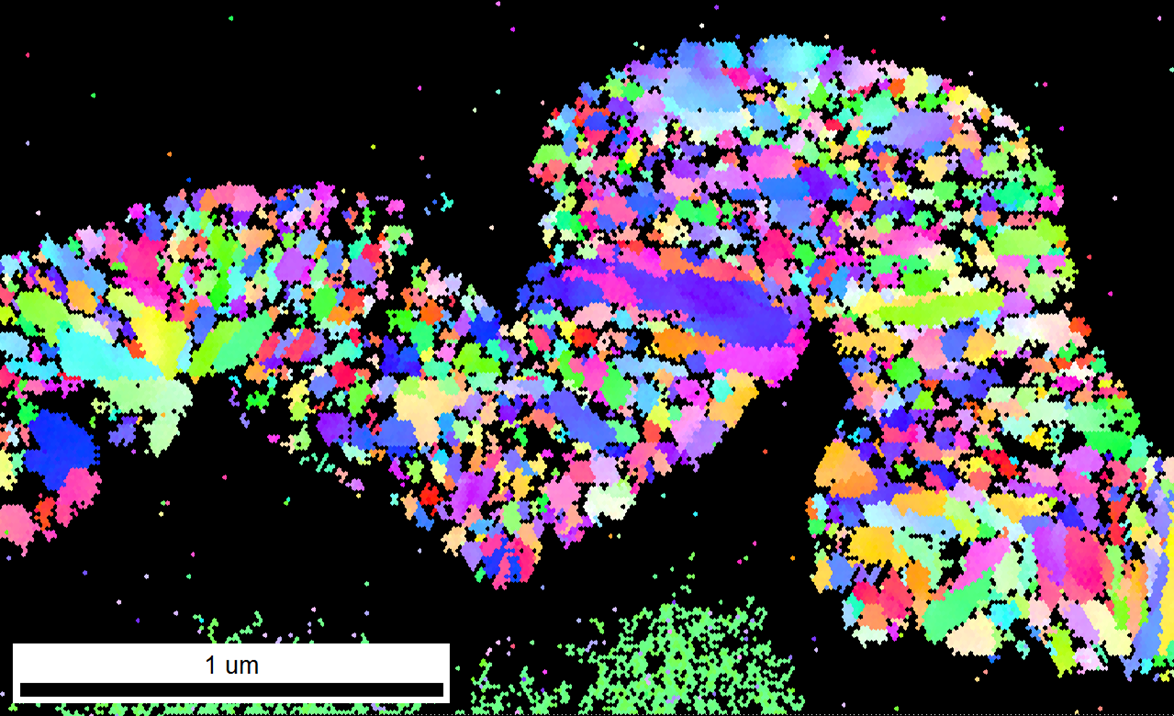


Figure S9. Cross-sectional electron backscatter diffraction (EBSD) image obtained for Type III Pt top-coated Pt/Ti electrode, which shows clearly that the main grains grow vertically along the Pt-Ti interface.

Table S2.The energy saving obtained for Type I Pt top-coated Pt/Ti electrode during the 1000-hour EHW electrolysis process.

|  | 1^st^ 250 hours | 2^nd^ 250 hours | 3^rd^ 250 hours | 4^th^ 250 hours |
| --- | --- | --- | --- | --- |
| Onset potential (V, vs RHE) | -6.2 | -5.77 | -5.84 | -6.2 |
| End potential (V, vs RHE) | -6.9 | -6.44 | -6.7 | -7.2 |
| Increased potential, *ΔE*  (mV hr^-1^) | 3.2 | | | |
| Increased energy consumption, P (kWh per kg of H_2_) | 85.7 | | | |

The energy saving, P at the cathode in kWh per kilogram of hydrogen gas is given by

$$P=\frac{i\Delta E(t/3600)}{i(t/F)}=\frac{\Delta EF}{3600}$$

The values of *ΔE* was calculated at current density from data in Table 1, and F constant is 96485.

1 Saleh, M. M. Electrochemical hydrogen evolution on polypyrrole from alkaline solutions. *Journal of Applied Electrochemistry* **30**, 939-944 (2000).
